# Emerging Multidrug‐Resistant Bacteria in Indonesian Neonatal Patients: Prevalence and Antimicrobial Resistance Profiles From Clinical Samples

**DOI:** 10.1155/ijm/2987105

**Published:** 2026-06-22

**Authors:** Muhammad Evy Prastiyanto, Afifah Khairunnisa, Andri Sukeksi, Tulus Ariyadi, Rizal Maarif Rukmana, Budi Santosa, Daniel Geleta

**Affiliations:** ^1^ Department of Medical Laboratory Technology, Faculty of Health and Nursing Science, Universitas Muhammadiyah Semarang, Semarang, 50273, Indonesia; ^2^ Research Center for Pharmaceutical Ingredients and Traditional Medicine, Research Organization for Health, National Research and Innovation Agency (BRIN), Bogor, 16911, Indonesia, brin.go.id; ^3^ Department of Epidemiology and Biostatistics, Jimma University, Jimma, Oromia, Ethiopia, ju.edu.et

**Keywords:** clinical samples, Indonesia, infections, MDR, neonatal sepsis

## Abstract

**Background:**

Neonatal sepsis poses a serious health challenge, especially with the increasing prevalence of multidrug‐resistant (MDR) bacteria among Indonesian neonates. This study examines the occurrence of MDR bacteria in clinical samples from neonates and analyzes their resistance patterns to enhance treatment strategies.

**Methods:**

From January 2022 to December 2024, a total of 223 bacterial isolates were gathered from neonatal patients with infections at Kanjeng Raden Mas Tumenggung Wongsonegoro Hospital in Semarang, Indonesia. The identification process commenced with Gram staining and assessment of colony morphology preceded by antibiotic susceptibility testing utilizing the VITEK2 Compact systems.

**Results:**

The study findings revealed a notable prevalence of MDR infections. Fecal samples accounted for 29.15% of the samples analyzed, with 63.1% exhibiting MDR characteristics. Blood samples, representing 24.66% of the samples, displayed a concerning MDR rate of 83.6%. Similarly, urine samples (24.66%) showed a MDR rate of 50.9%, while sputum samples (10.76%) presented a striking MDR classification of 91.7%. Key MDR rates were identified in *Escherichia coli* (45.6%), *Staphylococcus* spp. (70.4%), *Klebsiella pneumoniae* (71.9%), Acinetobacter spp. (63.6%), and *Pseudomonas aeruginosa* (100%). The overall prevalence of MDR infections was calculated at 66.40%, with significant resistance observed towards ampicillin (over 80%) in gram‐negative bacteria and benzylpenicillin (over 90%) in gram‐positive bacteria, particularly *Staphylococcus* spp.

**Conclusion:**

The findings underscore the urgent need for effective strategies to combat MDR infections in healthcare settings, emphasizing enhanced antibiotic management, tailored treatment protocols, and a more holistic and systematically structured research strategy for neonatal units in Indonesia.

## 1. Introduction

Neonatal sepsis continues to be a predominant cause of pediatric mortality worldwide, with its impact intensified by the swift increase in antimicrobial resistance (AMR) [1, 2]. Although AMR threatens health systems globally, it exerts a severe impact in Southeast Asia—particularly in Indonesia—where researchers consistently report some of the highest rates of multidrug‐resistant (MDR) infections [3, 4]. Indonesia, the world’s fourth most populous nation with almost 4.2 million births each year, has a significant problem in addressing newborn infections; nonetheless, published data regarding the prevalence and resistance profiles of MDR bacteria in Indonesian neonates are few [1].

One of the main contributors of MDR diseases is antibiotic resistance, which is a result of prolonged and inappropriate antibiotic use. Multiple studies explore natural product alternatives, from plants, mushrooms, marine bacteria, and lactic acid bacteria with inhibitory activity against MDR bacteria [5–9]. The most causal pathogens for newborn sepsis in Indonesian hospitals like other countries are gram‐negative bacteria, particularly *Klebsiella* spp. and *Acinetobacter* spp., which collectively represent over 50 percent of the cases [2, 10, 11]. Alarmingly, these pathogens exhibit alarming resistance to first‐line antibiotics, with susceptibility to WHO‐recommended regimens (e.g., ampicillin–gentamicin) as low as 25% [1]. The susceptibility of these pathogens to carbapenems, often considered drugs of last resort, is also declining, with *Acinetobacter* showing particularly low susceptibility rates (as low as 26%) [10]. Notably, the overall prevalence of MDR gram‐negative bacteria in neonatal intensive care units (NICUs) has been reported at over 60% in some Indonesian settings [11].

Despite the recognized threat, there are significant gaps in published data regarding the epidemiology, prevalence, and AMR profile of MDR bacteria in neonatal patients in Indonesia. Most previous studies have often been constrained by limited sample types, using only blood samples (neonatal sepsis), so there is a compelling need for updated, more comprehensive, cross‐sectional data regarding the prevalence and resistance profile of MDR bacteria among neonates in Indonesia using a variety of clinical samples. Consequently, this study aims to fill this gap by providing current epidemiologic data on MDR in neonatal patients, clarifying resistance patterns, and informing effective antimicrobial management and infection control strategies in this vulnerable population.

## 2. Materials and Methods

### 2.1. Study Design, Setting, and Period

This study used a descriptive cross‐sectional design to assess the prevalence and AMR patterns of MDR bacteria isolated from neonatal clinical specimens. MDR status in this study adhered to the standardized definition of multidrug resistance, whereby isolates were classified as MDR if they exhibited nonsusceptibility to at least one antimicrobial agent across three or more antimicrobial classes [12]. The study was conducted at KRMT Wongsonegoro Hospital, a tertiary referral hospital with a neonatal care unit, located in Semarang, Indonesia, from January 2022 to December 2024.

### 2.2. Study Population

The study included all neonatal patients under 28 days old admitted to the hospital with confirmed bacterial infections. Inclusion criteria were neonates from whom clinical samples were collected for bacterial culture during routine diagnostics. Exclusion criteria included samples with insufficient volume or those suspected of physical, chemical, or biological contamination. Biological contamination was defined as mixed growth of ≥ 2 organisms in normally sterile specimens or the isolation of typical commensals inconsistent with the clinical presentation. Samples collected after the initiation of antibiotic therapy, improperly labeled specimens, and those with inadequate transport or storage conditions were also excluded to ensure the accuracy and reliability of culture results. A total of 223 bacterial isolates from various clinical specimens were analyzed.

### 2.3. Data Collection Procedures

Clinical sample sources included fecal specimens collected directly from diapers using sterile techniques; sputum obtained via neonatal tracheal aspirates to minimize upper airway contamination; peripheral blood drawn aseptically through venipuncture into pediatric blood culture bottles; and urine collected using sterile pediatric collection bags. All specimens were handled under strict biosafety and aseptic conditions and were processed immediately upon receipt to maintain sample integrity, prevent degradation, and minimize the risk of environmental or procedural contamination. Initial bacterial identification was performed using Gram staining and evaluation of colony morphology on appropriate culture media. Subsequently, bacterial species identification and antimicrobial susceptibility testing were conducted using the automated VITEK2 Compact system (bioMérieux), following the manufacturer’s protocols [3, 4]. This system delivered standardized and reliable results for both bacterial identification and antibiotic resistance profiling, with appropriate positive and negatives controls in place.

### 2.4. Data Analysis

Descriptive statistics were utilized to summarize the prevalence of MDR bacteria across various clinical sample types and bacterial species. Frequencies and percentages were calculated for categorical variables, including bacterial species distribution and resistance rates to specific antibiotics. Antibiotic resistance profiles were analyzed to identify resistance patterns, with a focus on commonly used antibiotics in neonatal care. Data analysis was conducted using SPSS Version 26.0, ensuring a robust interpretation of prevalence and resistance trends.

### 2.5. Ethical Considerations

The study protocol received approval from the Institutional Review Board of Kanjeng Raden Mas Tumenggung (KRMT) Wongsonegoro Hospital. Informed consent was waived since the study used deidentified clinical isolates collected during routine care. Patient data confidentiality was strictly maintained, and all procedures followed ethical standards for research involving human subjects.

## 3. Results

### 3.1. Clinical Samples and MDR Bacteria Profiles

**FIGURE 1:**
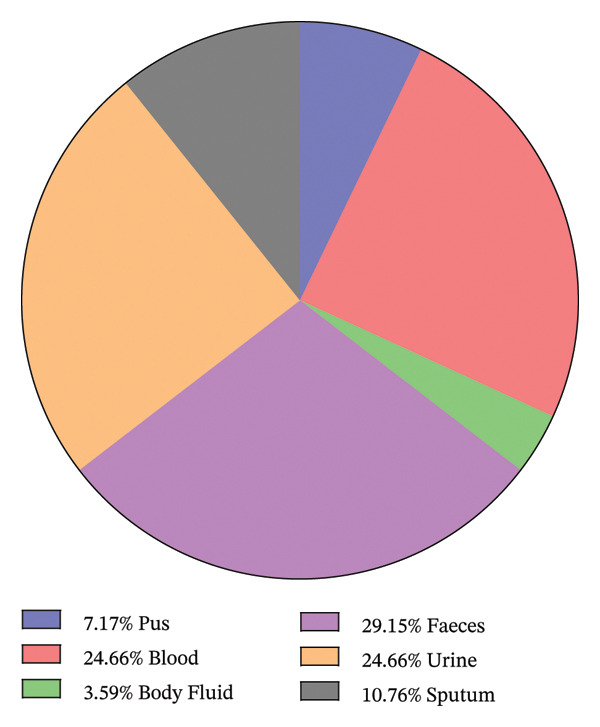
The percentage of clinical samples from neonatal patients (*N* = 223) is shown in the pie chart.

**FIGURE 2:**
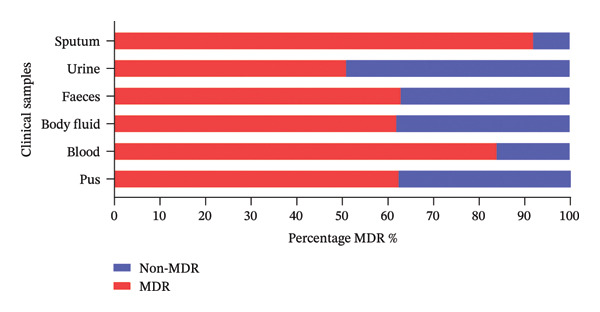
Bar graph depicting the percentage of MDR bacteria from clinical samples of neonatal patients (*N* = 223).

**FIGURE 3:**
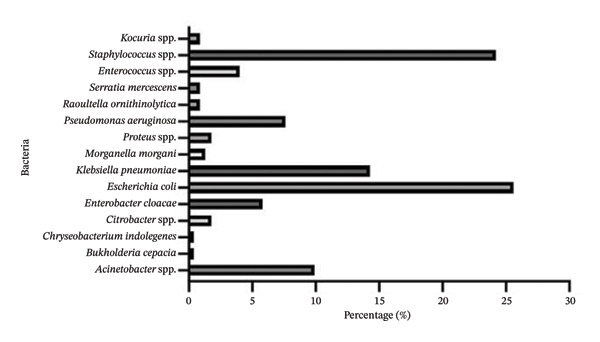
Bar graph depicting the bacterial spectrum of clinical samples of neonatal patients (*N* = 223).

A total of 223 bacterial isolates were recovered from clinical samples collected from infected neonatal patients. The distribution of isolates by sample type showed that faeces samples accounted for 29.15% (*n* = 65) of the total clinical specimens, with 63.1% of these showing MDR infections. Blood and urine samples each represented 24.66% (*n* = 55) of the total, with MDR infection rates of 83.6% and 50.9%, respectively (Figure 1). Sputum samples comprised 10.76% of the clinical samples and exhibited the highest MDR infection rate at 91.7%. Other sample types comprised the remaining 10.77% (*n* = 24) (Figure 2). The predominant strains included were *Escherichia coli, Staphylococcus* spp. *Klebsiella pneumoniae, Acinetobacter* spp., and *Pseudomonas aeruginosa* (Figure 3).

The overall prevalence of MDR bacterial isolates was 66.40% (*n* = 148/223). Among sample types, the proportion of MDR infections varied significantly (*p* < 0.01), with sputum samples exhibiting the highest MDR rate at 91.7%, followed by blood (83.6%), faeces (63.1%), and urine (50.9%) (Table 1). The data show a markedly higher proportion of multidrug resistance among gram‐negative bacteria compared to gram‐positive bacteria. The difference is statistically significant, as indicated by the *p* < 0.001 value.

**TABLE 1:** Proportion of MDR and non‐MDR isolates by gram classification, gender, and clinical sample source in Indonesia.

Bacteria classification	MDR (%)	Non‐MDR (%)	*p* *value*
Gram‐negative	99/158 (62.7)	59/158 (37.3)	*p* < 0.001
Gram‐positive	16/65 (24.6)	49/65 (75.4)	
Gender			*p* = 0.048
Boy	86/119 (72.3)	33/119 (27.7)	
Girl	62/104 (59.6)	42/104 (40.4)	
Clinical samples			*p* < 0.001
Pus	10/16 (62.5)	6/16 (37.5)	
Blood	46/55 (83.6)	9/55 (16.4)	
Body fluid	5/8 (62.5)	3/8 (37.5)	
Faeces	41/65 (63.1)	24/65 (36.9)	
Urine	28/55 (50.9)	27/55 (49.1)	
Sputum	22/24 (91.7)	2/24 (8.3)	

### 3.2. MDR Prevalence Among Bacterial Strains

**FIGURE 4:**
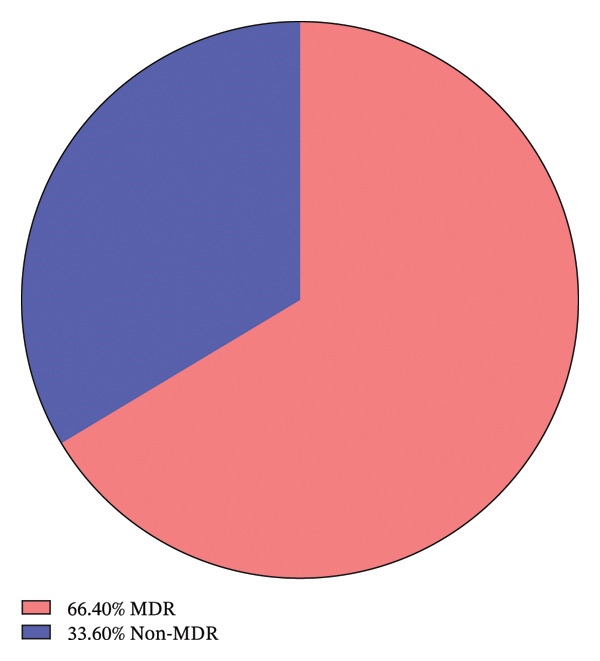
The percentage of MDR bacteria isolated from neonatal patients’ clinical samples is shown in the pie chart.

**FIGURE 5:**
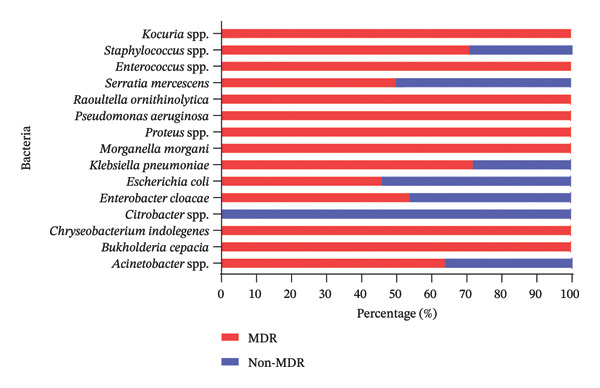
Bar graph showing the distribution of MDR bacterial species recovered from research participants.

The overall prevalence of MDR bacterial isolates in the study was 66.40% (Figure 4). Among the dominant species, MDR rates varied as follows: *Escherichia coli* showed 45.6% resistance, *Staphylococcus* spp. 70.4%, *Klebsiella pneumoniae* 71.9%, *Acinetobacter* spp. 63.6%, and *Pseudomonas aeruginosa* exhibited 100% MDR (Figure 5).

Resistance to antibiotics was notably high among gram‐negative bacteria, with ampicillin resistance exceeding 80% in the prevalent infectious species. For gram‐positive bacteria, particularly *Staphylococcus* spp., resistance to benzylpenicillin was observed at rates above 90%. Nondominant bacterial strains demonstrated very high MDR rates, with 100% resistance observed in *Burkholderia cepacia*, *Chryseobacterium indologenes*, *Morganella morganii*, *Proteus* spp., *Raoultella ornithinolytica*, *Enterococcus* spp., and *Kocuria* spp. (Table 2).

**TABLE 2:** Antibiotic resistance profiles of major bacterial pathogens isolated from neonates in Indonesia.

Antibiotics	*Acinetobacter spp*. (%)	*E. coli* (%)	*K. pneumoniae* (%)	*P. aeruginosa* (%)	*Staphylococcus spp*. (%)
Ampicillin	22/22 (100)	48/57 (84.2)	32/32 (100)	17/17 (100)	—
Ampicillin–sulbactam	14/22 (63.6)	35/57 (61.4)	26/32 (81.3)	17/17 (100)	—
Piperacillin	14/22 (63.6)	5/57 (8.8)	12/32 (37.5)	9/17 (52.9)	—
Cefazoline	22/22 (100)	27/57 (47.4)	25/32 (78.1)	17/17 (100)	—
Cefotaxime	19/22 (86.4)	23/57 (40.4)	22/32 (71)	17/17 (100)	—
Ceftazidime	15/22 (68.2)	13/57 (22.8)	23/32 (71.9)	9/17 (52.9)	—
Ceftriaxone	19/22 (86.4)	23/57 (40.4)	23/32 (71.9)	17/17 (100)	—
Cefepime	12/22 (54.5)	6/57 (10.5)	12/32 (37.5)	9/17 (100)	—
Aztreonam	22/22 (100)	18/57 (31.6)	23/32 (71.9)	11/17 (65.7)	—
Ertapenem	22/22 (100)	1/57 (1.8)	2/32 (6.3)	17/17 (100)	—
Meropenem	15/22 (68.2)	1/57 (1.8)	2/32 (6.3)	9/17 (52.9)	—
Amoxicillin	5/22 (22.7)	0/57 (0)	2/32 (6.3)	5/17 (29.4)	—
Gentamicin	12/22 (54.5)	16/57 (28.1)	20/32 (62.5)	7/17 (41.2)	20/54 (37)
Tigecycline	11/22 (50)	28/57 (49.1)	19/32 (59.4)	8/17 (47.1)	5/54 (9.3)
Nitrofurantoin	3/22 (13.6)	0/57 (0)	3/32 (9.4)	17/17 (100)	5/54 (9.3)
Trimethoprim sulfamethoxazole	22/22 (100)	3/57 (5.3)	21/32 (65.6)	17/17 (100)	21/54 (38.9)
Benzylpenicillin	—	—	—	—	51/54 (94.4)
Oxacillin	—	—	—	—	41/54 (75.9)
Ciprofloxacin	—	—	—	—	24/54 (44.4)
Levofloxacin	—	—	—	—	23/54 (42.6)
Moxifloxacin	—	—	—	—	20/54 (37)
Erythromycin	—	—	—	—	27/54 (50)
Clindamycin	—	—	—	—	28/54 (51.9)
Dalfopristin	—	—	—	—	3//54 (5.6)
Linezolid	—	—	—	—	3/54 (5.6)
Vancomycin	—	—	—	—	5/54 (9.3)

## 4. Discussion

Neonatal sepsis remains a critical cause of morbidity and mortality worldwide, particularly in low‐ and middle‐income countries such as Indonesia. Despite the recognized burden, previous studies on MDR bacterial infections in neonates within Indonesia have been limited in scope, often focusing solely on blood cultures from neonatal sepsis cases [1, 13]. This narrow focus has restricted understanding of the broader epidemiology and resistance patterns across different clinical sample types. Furthermore, existing data are often outdated or regionally confined, preventing comprehensive national‐level insights necessary for guiding empirical antibiotic therapy and stewardship programs. Our study addresses these gaps by providing a cross‐sectional, multisample analysis of MDR bacterial prevalence and resistance profiles in neonates from a major referral hospital in Semarang, Indonesia.

Our findings reveal a high overall prevalence of MDR bacteria (66.4%) among neonatal clinical isolates, with particularly alarming resistance rates in dominant pathogens such as *Escherichia coli* (45.6%), *Staphylococcus* spp. (70.4%), *Klebsiella pneumoniae* (71.9%), *Acinetobacter* spp. (63.6%), and *Pseudomonas aeruginosa* (100%). Notably, MDR prevalence was highest in sputum (91.7%) and blood samples (83.6%), underscoring the severity of respiratory and bloodstream infections in this vulnerable population. Resistance to commonly used antibiotics was also striking, with ampicillin resistance exceeding 80% among gram‐negative bacteria and benzylpenicillin resistance above 90% in gram‐positive *Staphylococcus* isolates. Additionally, nondominant strains such as *Burkholderia cepacia* and *Morganella morganii* exhibited 100% MDR rates, highlighting the emergence of less common but highly resistant pathogens.

Our finding of an overall MDR prevalence of 66.4% aligns with global concerns about rising MDR rates in NICUs. The analysis of the research findings reveals a significantly higher prevalence of MDR gram‐negative bacteria (62.7%) compared to gram‐positive bacteria (24.6%), with a statistically significant difference (*p* < 0.001). This aligns with global patterns of antibiotic resistance and provides specific insights into microbial adaptation and clinical challenges. For instance, studies from Southeast Asia and Ethiopia report MDR rates ranging from 50% to 70% and 88.4% in neonatal pathogens, respectively [2, 14]. Gram‐negative had a two times higher chance of being MDR [3]. The high MDR rates observed in dominant strains such as *Escherichia coli* (45.6%), *Staphylococcus* spp. (70.4%), *Klebsiella pneumoniae* (71.9%), and *Acinetobacter* spp. (63.6%) are consistent with findings from other regional studies that have highlighted these pathogens as major contributors to neonatal morbidity and mortality [15, 16] like in other regions of the world [17]. The 100% MDR rate in *Pseudomonas aeruginosa* is particularly alarming and suggests the emergence of extensively drug‐resistant strains, a trend also noted in recent NICU surveillance reports [18].

Particularly, the 66.4% overall prevalence of MDR bacteria among neonatal clinical isolates in our study is notably higher than most reports from neighboring countries, highlighting a disproportionate MDR burden in our setting [19, 20]. Evidence from Southeast Asia, other LMICs, Pakistan, China, India, Iran, and Egypt shows MDR prevalence similar to or exceeding our finding, including a study in Cairo reporting 91% MDR isolates in pediatric ICUs [19], whereas a study from Amman, Jordan, documented lower resistance to antibiotics such as penicillin, cefotaxime, cefoxitin, and oxacillin in NICUs [21]. Pathogen‐specific comparisons indicate that the 45.6% MDR *Escherichia coli* observed here is lower than some LMIC reports where resistance to ampicillin and gentamicin is frequently very high [19]; the 70.4% MDR *Staphylococcus* spp. is comparable to other findings though MRSA prevalence varies widely [20, 22]; and the 71.9% MDR *Klebsiella pneumoniae* aligns with consistently high resistance rates to third‐generation cephalosporins and carbapenems reported elsewhere [23]. The 63.6% MDR *Acinetobacter spp.* is substantial but lower than the 78% carbapenem resistance reported in India [19], while the 100% MDR *Pseudomonas aeruginosa* is alarming but difficult to compare due to limited regional data. The > 80% ampicillin resistance among gram‐negative isolates and > 90% benzylpenicillin resistance among *Staphylococcus* spp. correspond with reports from many developing countries [24, 25]. Overall, variations in MDR prevalence across studies likely reflect differences in geography, hospital settings, diagnostic capacity, study periods, and infection control practices [20, 25].

Notably, resistance to ampicillin among gram‐negative bacteria and benzylpenicillin among gram‐positive isolates observed here corroborates earlier reports indicating widespread resistance to first‐line antibiotics in neonatal infections [24]. However, the inclusion of nondominant strains such as *Burkholderia cepacia* and *Chryseobacterium indologenes* with 100% MDR rates is a novel finding that expands the known spectrum of neonatal MDR pathogens in Indonesia and underscores the complexity of the neonatal microbiome and infection landscape.

A major strength of this study is its cross‐sectional design spanning 3 years and encompassing a diverse array of clinical samples, which enhances the generalizability and robustness of the findings. Utilizing the VITEK2 Compact system for both identification and susceptibility testing provided standardized, reliable data critical for guiding empirical therapy. The inclusion of multiple sample types beyond blood allowed detection of MDR pathogens in sites less commonly studied in neonates, such as feces and sputum, revealing high MDR infection rates (63.1% and 91.7%, respectively) that may reflect colonization or infection reservoirs previously underappreciated in neonatal care and epidemiological significance of MDR organisms in neonates. These findings indicate several shortcomings in the hospital’s current neonatal anti‐infective protocol, suggesting that existing practices may not adequately detect, prevent, or manage MDR organism circulation within the neonatal unit.

One unexpected finding was the extraordinarily high MDR rates in nondominant strains, all reaching 100%. This suggests that less commonly isolated bacteria may harbor extensive resistance mechanisms, potentially serving as hidden reservoirs for resistance genes that could be transferred to more prevalent pathogens. This finding highlights the need for ongoing surveillance beyond the usual suspects and may prompt reconsideration of infection control policies to address these emerging threats. Additionally, the high MDR prevalence in fecal samples raises important questions about the role of the neonatal gut microbiota as a reservoir for resistant bacteria, which could have implications for horizontal gene transfer and subsequent infections. This observation aligns with emerging evidence linking gut colonization with MDR organisms to increased risk of invasive infections in neonates [15].

While this study provides comprehensive data on MDR prevalence, several limitations should be acknowledged. The single‐center design may limit generalizability to other Indonesian regions with differing healthcare settings and antibiotic usage patterns. Additionally, the cross‐sectional nature precludes assessment of temporal trends or causality between antibiotic exposure and resistance development. The reliance on VITEK2 Compact for identification and susceptibility testing, while standard, may miss some resistance mechanisms detectable only by molecular methods. Finally, clinical outcome data were not included, limiting the ability to correlate resistance patterns with patient prognosis or treatment efficacy. Moreover, this study highlights interconnected gaps within the hospital’s neonatal anti‐infective protocol, infection‐control practices, and antimicrobial stewardship systems [26]. The persistently high resistance rates—such as > 80% resistance to ampicillin [27]—demonstrate continued reliance on outdated ampicillin‐based empiric regimens that no longer correspond to local susceptibility profiles. Nearly half of neonatal sepsis pathogens are resistant to first‐line agents like ampicillin, underscoring the clinical implications of this misalignment [28]. At the same time, the lack of routine colonization surveillance allows hidden reservoirs of MDR organisms in fecal and respiratory sites to go undetected, reducing opportunities for early intervention and targeted infection‐control measures—particularly in high‐risk preterm neonates [26, 28]. The elevated MDR prevalence further reflects inconsistent adherence to antimicrobial stewardship principles, including oversight of antibiotic selection and treatment duration [27, 28]. Addressing these integrated shortcomings will require aligning empirical guidelines with current resistance data, strengthening infection‐control strategies, and embedding regular surveillance review into annual policy updates to maintain responsiveness to evolving neonatal MDR trends [26, 27].

Future research should expand to multicenter, longitudinal studies to capture regional variability and temporal dynamics of MDR bacterial infections in neonates. Incorporating molecular diagnostics and genomic surveillance would enhance the detection of resistance genes and transmission pathways, informing targeted interventions. Investigating the impact of antibiotic stewardship programs and infection control measures on MDR prevalence in neonatal units is also critical. Moreover, clinical studies assessing treatment outcomes based on resistance profiles can guide optimization of empirical therapy protocols. Finally, exploring the role of colonization and environmental reservoirs in neonatal MDR infections could provide insights for preventive strategies.

### 4.1. Limitations of the Study

This study has several limitations that should be acknowledged. First, the research was conducted at a single tertiary hospital, which may limit the generalizability of the findings to other regions of Indonesia with different patient populations, healthcare practices, and antibiotic‐use patterns. Second, although the study included a broad range of clinical samples, it did not incorporate clinical characteristic data of the neonates—such as gestational age, birth weight, comorbidities, prior antibiotic exposure, or use of invasive devices. The absence of these variables restricts epidemiological interpretation and prevents meaningful correlation between microbiological findings and clinical context, limiting conclusions about the clinical significance and risk factors associated with MDR infections.

Third, the cross‐sectional design provides a snapshot of MDR prevalence but does not allow assessment of temporal trends, causality, or the impact of antibiotic stewardship initiatives. Additionally, the study relied on automated phenotypic methods (VITEK2 Compact), which, while standardized and reliable, may fail to detect certain resistance mechanisms that require molecular or genomic testing. Finally, clinical outcome data—such as mortality, length of hospital stay, or treatment response—were not available, preventing evaluation of the direct clinical impact of MDR infections on neonatal health outcomes.

## 5. Conclusion

In conclusion, this study fills a significant knowledge gap by providing updated, comprehensive data on the prevalence and AMR profiles of MDR bacteria in Indonesian neonates across multiple clinical samples. The high burden of MDR pathogens, particularly in blood and respiratory samples, and widespread resistance to commonly used antibiotics underscore the urgent need for improved antimicrobial stewardship and tailored treatment regimens in neonatal care. Our findings serve as a critical resource for clinicians and policymakers aiming to combat neonatal infections and reduce the impact of AMR in Indonesia and similar settings. However, the absence of neonatal clinical data, the single‐center study design, reliance on phenotypic methods, and the lack of clinical outcome measures require that the findings be interpreted with caution. Future research should incorporate detailed clinical variables, expand surveillance to multiple facilities, employ molecular resistance testing, and include longitudinal and outcome‐focused analyses to better elucidate the clinical significance and impact of MDR infections in neonates.

## Author Contributions

All authors contributed equally to this study across various aspects of the research process. They collaborated on conceptualization, data curation, and formal analysis, ensuring a comprehensive approach to the investigation. Each author played a pivotal role in methodology development, project administration, and securing funding. Additionally, they equally participated in software use and validation of findings. Contributions to visualization and the writing process, including the original draft and subsequent reviews, were also shared equally among the authors, reflecting their collective commitment to the integrity and quality of the research.

## Funding

No specific funding source was utilized for this study.

## Conflicts of Interest

The authors declare no conflicts of interest.

## Data Availability

The data used to support the findings of this study are included within the article.
